# Solitary Uncommon Metastasis in Non-Small Cell Lung Cancer

**DOI:** 10.3390/reports6010008

**Published:** 2023-02-15

**Authors:** Hyung Keun Cha, Jun Hyeok Lim, Woo Kyung Ryu, Lucia Kim, Jeong-Seon Ryu

**Affiliations:** 1 Division of Pulmonology, Department of Internal Medicine, Inha University Hospital, Inha University School of Medicine, Incheon 22332, Republic of Korea; 2 Department of Pathology, Inha University Hospital, Inha University School of Medicine, Incheon 22332, Republic of Korea

**Keywords:** non-small-cell lung cancer, case series, tail of the pancreas, thyroid gland, right back and breast

## Abstract

The major sites of metastasis in non-small cell lung cancer (NSCLC) are bones, the brain, adrenal glands, the liver, the contralateral lung, and distant lymph nodes. Solitary metastasis in an uncommon site is very rare; therefore, it has not often been reported. Identifying whether a solitary lesion is a metastatic lesion is important because it decisively influences the stage and treatment decisions. We report a series of cases of NSCLC diagnosis with uncommon solitary metastasis. (1) A 71-year-old man was diagnosed with poorly differentiated NSCLC after a bronchoscopic biopsy of a tumor in the bronchus of the right middle lobe. A hypermetabolic lesion was observed in the tail of the pancreas using positron emission tomography/computed tomography (PET/CT), and metastasis of NSCLC was confirmed using endoscopic ultrasound fine-needle aspiration (EUS-FNA). (2) A 77-year-old man was diagnosed with squamous cell carcinoma after a bronchoscopic biopsy of a tumor in the bronchus of the left upper lobe. A hypermetabolic lesion was observed in the bilateral lobes of the thyroid gland using PET/CT, and metastasis of the squamous cell carcinoma was confirmed by FNA and cytology. (3) A 79-year-old woman was diagnosed with adenocarcinoma by brushing cytology performed on the apicoposterior segmental bronchus of the left upper lobe. Hypermetabolic lesions were observed using PET/CT in the subcutaneous layer of the right back and the left breast, and metastases of adenocarcinoma were confirmed by biopsies in each lesion. These three patients were treated with platinum-based chemotherapy for stage IV lung cancer. With this case series, we recommend that, when a solitary lesion is observed in NSCLC patients, a tissue biopsy should be performed, even if the lesion is located in an organ where lung cancer metastasis is uncommon.

## 1. Introduction

Cancer metastasis is responsible for 90% of cancer-related deaths [1]. Unfortunately, approximately forty percent of non-small-cell lung cancer (NSCLC) patients present with metastasis at the time of diagnosis [2]. The major sites of NSCLC metastasis include the brain, bones, adrenal glands, liver, contralateral lungs, and distant lymph nodes [3]. Metastasis to other organs is uncommon (less than 5%), and solitary metastases in these uncommon sites have rarely been reported [4,5,6]. Therefore, without a biopsy, clinicians often regard lesions in uncommon sites as benign or double primary cancer [7]. However, it is important to identify whether a solitary lesion is a metastatic lesion because it decisively influences the stage and treatment decision. Traditionally, that panel of immunohistochemistry stains can provide sufficient information for the differential diagnosis. Nowadays, due to advances in molecular pathology, specific genomic alterations, such as epidermal growth factor receptor (EGFR) mutations, can provide additional diagnostic evidence to determine the origin of the cancer cells. The treatment is often controversial when used for uncommon solitary metastasis. Local therapy is frequently considered in such settings, although uncommon metastases are classified as M1b and stage IV based on the non-small-cell lung cancer staging system. Here, we report on patients diagnosed with NSCLC with solitary metastasis to organs, such as the tail of the pancreas, the thyroid gland, and the right back and breast, where metastasis is uncommon. We present the following cases in accordance with the case-series reporting checklist.

## 2. Case Presentation

We retrospectively reviewed 974 patients diagnosed with NSCLC at Inha University Hospital (Incheon, Republic of Korea) from January 2005 to December 2018. All patients were diagnosed with stage IV NSCLC by imaging tests, including computed tomography (CT), positron emission tomography (PET), whole-body bone scans, and brain magnetic resonance imaging (MRI), at the time of diagnosis. Patients who did not undergo brain MRI (n = 23) or a whole-body bone scan (n = 15) or PET (n = 28) were excluded. All patients underwent a standard staging work-up at the Inha University hospital. Information including the patients’ gender, Eastern Cooperative Oncology Group (ECOG) performance status, smoking history, T category, N category, number and location of metastatic organs, histology, and EGFR or ALK mutation status were analyzed. The stages of all patients were evaluated using the eighth edition of the TNM staging system. All information was prospectively collected from the electronic medical records of the Inha Lung Cancer Cohort of Inha University Hospital.

Distant metastasis was identified based on the results of 18F-fluorodeoxyglucose positron emission tomography/computed tomography (FDG-PET/CT) scanning. All patients had fasted for at least six hours, and their fasting blood glucose levels were <150 mg/dL prior to the FDG-PET/CT scan. PET/CT scans were conducted using a Biograph Duo (Siemens Medical Solutions USA, Inc.) and a PET/CT scanner (Discovery PET/CT 690 and a 64-slice CT, GE Healthcare). The parameters for the CT attenuation correction acquisition of the GE scanner were as follows: tube current—100 to 200 mA Auto mA: ASiR; and slice thickness—3.75 mm; voltage—120 kV. The parameter settings for the Siemens device were: CARE Dose 4D mA tube current; and slice thickness—5.00 mm; voltage—130 kV. PET data were obtained using 2 min/bed position with a 128 × 128 matrix. The whole-body emission scans were obtained using a PET camera 60 minutes after the injection of FDG. The acquired images were reconstructed using the iterative reconstruction algorithm. All PET images were adjusted for attenuation using the acquired CT data. All FDG-PET/CT images for the initial staging work-up were evaluated by two experienced physicians, specialists in nuclear medicine, who did not have any knowledge of either the imaging results or the clinical data. Abnormal FDG uptake was determined when the accumulation of the radiotracer moderately to markedly increased, compared to the expected uptake in normal anatomic structures or surrounding tissues, with the exclusion of urinary and physiologic bowel activity.

The median age of the 902 patients with metastatic NSCLC at the time of diagnosis was 70 years (range, 34–96). Of these patients, 628 (69.6%) had adenocarcinoma and 185 (20.5%) had squamous cell carcinoma. Regarding the organ of metastasis, bone was most common (388 patients, 43.0%), followed by lung (326 patients, 36.1%), brain (238 patients, 26.4%), adrenal gland (120 patients, 13.3%), abdominal lymph nodes (105 patients, 11.6%), and liver (105 patients, 11.6%). Among the 902 patients with stage IV lung cancer with metastasis, 409 patients (45.3%) had solitary metastasis, and only 13 showed solitary uncommon metastasis. Among these patients, we report a case series of three patients with a solitary metastasis in an uncommon site at diagnosis (Table 1). The authors are accountable for all aspects of the work and ensured that questions related to the accuracy or integrity of any part of the work were appropriately investigated and resolved. All procedures undertaken in this study were performed in accordance with the ethical standards of the institutional and/or national research committees, and with the Helsinki Declaration (as revised in 2013). This study (ID 2020-03-018-001) has approval from the Inha University Hospital institutional review board. Informed consent was obtained from all patients involved in the study. 

### 2.1. Patient 1

**Figure 1:**
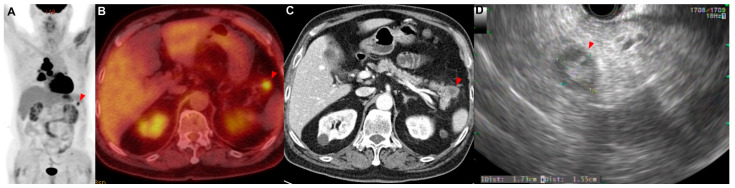
Patient 1: a 71-year-old man diagnosed with non-small-cell lung cancer (NSCLC) with a solitary metastasis in the tail of the pancreas. (**A**–**C**) Positron emission tomography/computed tomography (PET/CT) showed a solitary metastatic lesion (red arrowhead) in the tail of the pancreas. (**D**) EUS-FNA was performed at the metastatic lesion (red arrowhead) in the pancreas.

**Figure A1:**
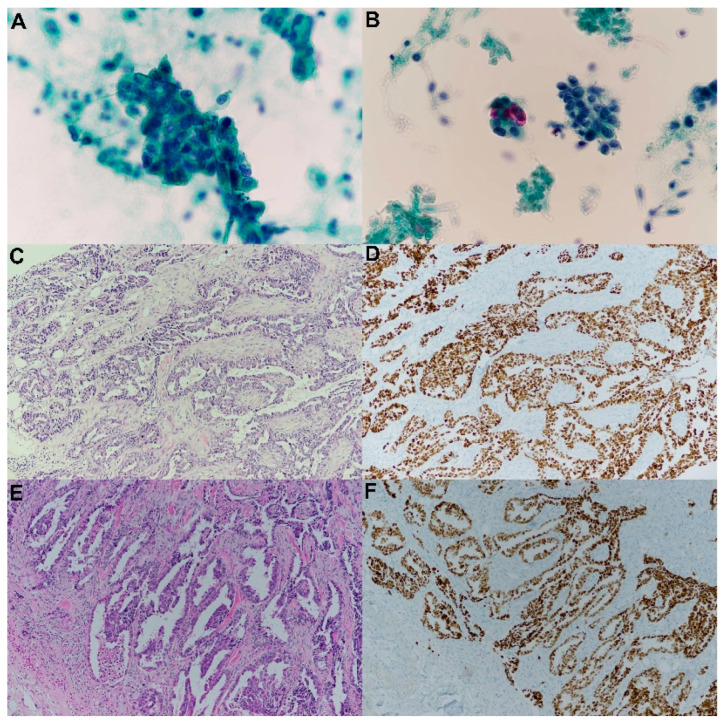
Pathology images of metastatic lesions. (**A**) Cytology of pancreatic metastasis, (**B**) cytology of thyroid metastasis, (**C**) hematoxylin and eosin (H&E) staining of breast metastasis, (**D**) thyroid transcription factor (TTF-1) staining of breast metastasis, (**E**) H&E staining of metastatic lesions in the subcutaneous layer of the right back, and (**F**) TTF-1 staining of metastatic lesions in subcutaneous layer of the right back.

A 71-year-old man presented with a 2-week history of progressive dyspnea and chest discomfort. He had a history of 30 pack years of smoking and underwent surgery for cerebral hemorrhage 7 years ago. He stated that he did not take any medications or have other illnesses, including cardiovascular, allergic, rheumatic, or respiratory conditions. A contrast-enhanced computed tomography (CT) scan of his chest showed a lung mass invading the lobar bronchus of the right middle lobe. A bronchoscopic biopsy was performed, and poorly differentiated NSCLC was diagnosed. If there was no metastasis, the tumor was considered stage IIIB as T4N2. There was no suspicious finding of distant metastasis except for a hypermetabolic lesion (SUVmax = 4.76) of the pancreas tail, which was observed using positron emission tomography/computed tomography (PET/CT) (Figure 1A–C). Aspiration cytology was performed from the lesion of the pancreas tail through endoscopic ultrasound-fine needle aspiration (EUS-FNA) (Figure 1D). As a result of the cytological examination, a poorly differentiated non-small-cell carcinoma with the same morphology as the cancer cells in the lung cancer tissue was confirmed in the aspirated specimen (Figure A1A). This patient was treated with six courses of platinum-based chemotherapy (gemcitabine and cisplatin) for stage IV NSCLC.

### 2.2. Patient 2

**Figure 2:**
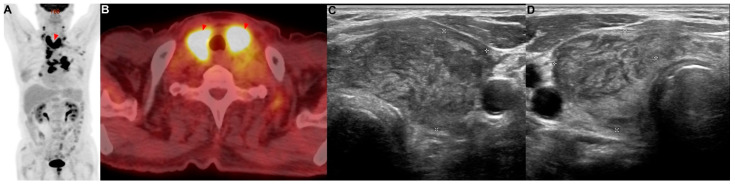
Patient 2: a 77-year-old man diagnosed with squamous cell carcinoma of the lung with metastases in the thyroid. (**A**,**B**) PET/CT showed metastatic lesions (red arrowhead) in the bilateral lobes of the thyroid. (**C**,**D**) Metastatic lesions were observed in the (**C**) left and (**D**) right thyroid.

A 77-year-old man with a medical history of diabetes mellitus was referred for an abnormal chest radiograph, which was taken at a public health clinic. He had a history of 57 pack years of smoking. He stated that he did not take any medications or have any illnesses, including cardiovascular, allergic, rheumatic, or respiratory conditions. A contrast-enhanced CT scan of the chest showed a lung mass invading a lobar bronchus of the left upper lobe. A bronchoscopic biopsy was performed and squamous cell carcinoma was diagnosed. If there was no metastasis, the tumor was considered stage IIIC as T4N3. There was no suspicious finding of distant metastasis except for a hypermetabolic lesion (SUVmax = 13.42) of the bilateral lobes of the thyroid gland in PET/CT (Figure 2A,B). FNA and cytology were performed on the lesion of the left thyroid gland (Figure 2C,D), and metastasis of squamous cell carcinoma was confirmed (Figure A1B). This patient was treated with six courses of platinum-based chemotherapy (gemcitabine and cisplatin) for stage IV squamous cell carcinoma.

### 2.3. Patient 3

**Figure 3:**
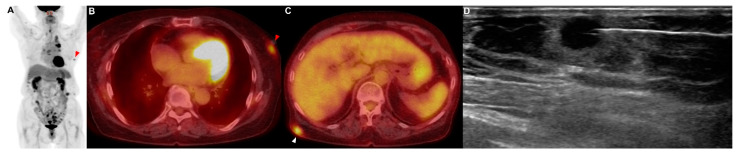
Patient 3: a 79-year-old woman diagnosed with lung adenocarcinoma with metastases in subcutaneous tissue. (**A**–**C**) The PET/CT showed a metastatic lesion (red arrowhead) in the left breast. I PET/CT showed a metastatic lesion (white arrowhead) in the subcutaneous layer of the middle part of the right back. (**D**) The core needle biopsy was performed on the lesion of the left breast.

A 79-year-old woman who had never smoked presented with 2 weeks of hoarseness and a history of hypertension and diabetes mellitus. She stated that she had no other illnesses, including cardiovascular, allergic, rheumatic or respiratory conditions. A contrast-enhanced CT scan of her chest showed that a lung mass originating in the left upper lobe had invaded the mediastinum. No endobronchial tumor was observed on the bronchoscopy, and a narrowing of the apicoposterior segmental bronchus of the left upper lobe was observed. Adenocarcinoma was confirmed by brushing cytology of the apicoposterior segmental bronchus of the left upper lobe. If there was no metastasis, the tumor was considered stage IIIB as T4N2. On the PET/CT scan, hypermetabolic lesions were observed in the subcutaneous layer of the middle part of the right back (SUVmax = 4.64) and in the left breast (SUVmax = 3.52) (Figure 3A–C). A core-needle biopsy was performed on the lesion in the left breast (Figure 3D), and the pathological examination reported metastasis of lung adenocarcinoma (TTF-1: positive; GATA-3: negative) (Figure A1C,D). In addition, an excision biopsy was performed on the lesion of the right back, and the pathological examination showed metastasis of the lung adenocarcinoma (TTF-1: positive; ALK: negative; GATA-3: negative; CDX-2: negative) (Figure A1E,F). Additionally, EGFR 19 deletion mutation was detected in this metastatic tissue using an EGFR PCR test. Therefore, this patient was treated with an EGFR tyrosine kinase inhibitor for EGFR-mutated-stage-IV adenocarcinoma.

## 3. Discussion

Several case reports and studies have investigated uncommon metastases of lung cancer. However, these reports and studies have only slightly improved our knowledge about uncommon metastases of lung cancer. Here, we report a series of cases of solitary metastasis of NSCLC in uncommon sites. Patients with uncommon metastases of NSCLC have a worse prognosis than those with common metastases of NSCLC [7]. Therefore, prompt and aggressive treatment is required for these patients. Generally, metastasis to an uncommon site is observed in the terminal stage as part of a diffuse metastatic process. Uncommon metastasis is rarely observed as a solitary lesion in NSCLC patients. Our report suggests that lung cancer metastasis should be considered when a solitary lesion with increased SUVmax is observed in a site where lung cancer does not typically metastasize.

About 15% of patients initially classified as stage I-III NSCLC by CT scan are reclassified as stage IV with a PET scan [8]. According to previous reports, PET/CT could lead to stage migration and a modification of the treatment in about 20–25% of the patients with NSCLC. In addition, PET/CT is an accurate means of determining information about distant metastatic disease in apparently nonmetastatic NSCLC patients. Therefore, all patients should undergo evaluation by a whole-body PET scan to detect metastatic lesions at diagnosis. If an accurate evaluation of distant metastases is not performed, only local treatment for lung lesions may be administrated. However, previous studies reported that patients who received systemic and local treatment had better outcomes than those who received only local treatment [9,10].

A few case reports have discussed solitary metastasis to organs where lung cancer metastasis is uncommon, such as the stomach and spleen [4,5,6]. In our case series, which presents a variety of cases of uncommon metastases, we suggest a clinical approach for patients with suspected metastases in uncommon sites. In two of the three patients (patients 1 and 2), FNA and cytologic examination were performed at the metastatic lesion. Fortunately, lung cancer metastasis was determined by observing a cell type that does not typically appear in metastatic organs.

However, in many cases, it is difficult to differentiate between primary and metastatic cancer using only cytological examination. For example, in a patient with lung adenocarcinoma, if metastasis is suspected in certain locations where adenocarcinoma commonly occurs as primary cancer, such as the colon or breast, it may be difficult to differentiate primary cancer from metastasis by cytology alone. Therefore, clinicians should determine whether tissue biopsy and immunohistochemistry are necessary to confirm metastasis by considering the histologic type of the lung cancer and the organ with the suspected metastasis. In patient 3, immunohistochemistry with tissue biopsy was essential in identifying the metastasis of lung cancer [11].

Although this case series recommends histological confirmation with a biopsy of a suspected lesion of solitary metastasis, this should be interpreted cautiously. In some cases, a suspected lesion of solitary metastasis is observed in a site that is difficult to access for biopsy. In this case, clinicians should carefully consider the risks and benefits of an aggressive biopsy.

## 4. Conclusions

In conclusion, when a solitary lesion is observed in NSCLC patients, a tissue biopsy should be performed, even if the lesion is located in an organ where lung cancer metastasis is uncommon. This case series systematically presented cases of solitary metastasis in uncommon sites for metastasis. Further research should be conducted.

## Figures and Tables

**Table 1:** Characteristics of three patients.

	Patient 1	Patient 2	Patient 3
Age	71	77	79
Gender	Male	Male	Female
Smoking	Current smoker	Ex-smoker	Never smoker
Stage (without metastasis)	IIIB	IIIC	IIIB
Site of metastasis	Pancreas	Thyroid	Subcutaneous tissue/breast
Diagnostic method of primary tumor	Bronchoscopic biopsy	Bronchoscopic biopsy	Bronchoscopic brushing cytology
Diagnostic method of metastasis	EUS-FNA	FNA	Excision biopsy/core needle biopsy
Histology of primary tumor	Poorly differentiated NSCLC	Squamous cell carcinoma	Adenocarcinoma
Histology of metastasis	Poorly differentiated NSCLC	Squamous cell carcinoma	Adenocarcinoma/adenocarcinoma

EUS, endoscopic ultrasound; FNA, fine-needle aspiration, NSCLC, non-small-cell lung carcinoma.

## Data Availability

The data used in this case report are available upon reasonable request from the corresponding author.
